# Comparison of Corticosterone Concentrations in Dermal Secretions and Urine in Free-Ranging Marine Toads (*Rhinella marina*) in Human Care

**DOI:** 10.1155/2023/1467549

**Published:** 2023-09-19

**Authors:** Emma Clarke, Kimberly Ange-van Heugten, Troy N. Tollefson, Frank N. Ridgley, Dustin Smith, Janine L. Brown, Heather Scott, Larry J. Minter

**Affiliations:** ^1^Department of Clinical Sciences, North Carolina State University, College of Veterinary Medicine, 1060 William Moore Dr., Raleigh, NC 27607, USA; ^2^Department of Animal Science, North Carolina State University, 120 W. Broughton Dr., Raleigh, NC 27695, USA; ^3^Environment Medicine Consortium, North Carolina State University, 1060 William Moore Dr., Raleigh, NC 27607, USA; ^4^Mazuri® Exotic Animal Nutrition, PMI Nutrition, 4001 Lexington Ave. North, Arden Hills, MN 55126, USA; ^5^The Conservation and Research Department, Zoo Miami, 12400 SW 152nd St., Miami, FL 33177, USA; ^6^North Carolina Zoo, 4401 Zoo Pkwy, Asheboro, NC 27205, USA; ^7^Smithsonian Conservation Biology Institute, 1500 Remount Rd., Front Royal, VA 22630, USA

## Abstract

Corticosterone concentrations have been measured in amphibians by collecting blood or urine samples. However, blood sampling is invasive, and urine can be difficult to collect. A novel method of swabbing the skin of an amphibian has been utilized in numerous species but has not been verified in marine toads (*Rhinella marina*). This pilot study tested dermal swabs as a noninvasive method for collecting and measuring dermal corticosterone secretions. Swabs were used to collect dermal secretion samples from sixty-six free-ranging marine toads collected on Zoo Miami grounds. The subsequent day the toads were shipped to the North Carolina Zoo where dermal samples were collected again. Additional dermal and urine samples were collected on days 9, 15, 32, and 62 under human care to measure corticosterone concentrations. There was no significant correlation (*P* ≥ 0.05) noted between corticosterone concentrations reported in dermal swabs and those in urine samples at all four of the euthanasia time points or between the corticosterone concentrations reported in either urine or dermal swabs and the weight of the toads. Dermal swab concentrations (ng/mL) were significantly higher (*P* ≤ 0.05) on the day of capture (0.64 ± 0.03) and the day of arrival (0.67 ± 0.03) than on day 15 (0.47 ± 0.03). The urine corticosterone concentrations decreased while the toads were in human care with a significant decrease (*P* ≤ 0.05) between days 9 (0.45 ± 0.07) and 32 (0.21 ± 0.06). This study demonstrated that dermal swabs can be used to collect marine toad corticosterone concentration samples.

## 1. Introduction

Determining the health and viability of an ecosystem has been a topic of research for many decades [1, 2]. It is well documented that the diversity of species, specifically the presence of amphibians, is a good indicator of the overall environmental health and can be applied to ecosystems across the globe [3, 4]. Many studies have postulated that we are currently living through a mass extinction of all species across multiple taxonomic classes with amphibian species facing an extinction rate, i.e., 211 times higher than other nonamphibian species [5–7]. This global decline in amphibians is believed to be the result of both natural causes, such as the chytrid fungus *Batrachochytrium dendrobatidis* [5, 8–10] and those linked to human interference, including pollution [11, 12], habitat destruction [13, 14], and the introduction of invasive species [15]. These changes can affect individuals ranging from the population level to the molecular level [16].

There is no straightforward method to quantify the biodiversity of an ecosystem. The simplest index that has been used traditionally is the species richness index, which counts the number of unique animal and plant species in a given geographical area [17]. The trouble with this index is that it does not consider the distribution of the species and the abundance of individual species. In addition, many animal and plant species must die to quantify a decrease in biodiversity. Researchers are now trying to develop methods to assess the health of an ecosystem by measuring the stress of amphibians via their concentration of circulating corticosterone to test the hypothesis that environments that have symptoms of distress syndrome will be inhabited by chronically stressed animals [18, 19].

In vertebrates, stress can be evaluated by measuring the concentration of circulating glucocorticoids [20–23]. The hypothalamus-pituitary-interrenal axis (HPI) releases hormones that ultimately stimulate the release of corticotropin from the pituitary gland into the circulatory system [24]. In amphibians, corticotropin goes on to stimulate the interrenal gland, which is the amphibian equivalent of the adrenal cortex, to produce corticosterone, the primary corticosteroid of amphibians [24, 25]. The main purpose of corticosterone is to promote gluconeogenesis so that the stored energy can be converted and utilized for the body [26]. For example, corticosterone concentrations naturally oscillate with the circadian rhythm of an individual [27–29], which for free-ranging marine toads peaks during the late evening and the early morning when they are the most active [27]. Increased corticosterone concentrations have also been documented during other innocuous life history events such as mating seasons in marine toads [30]. However, corticosterone is also released in response to a myriad of scenarios including periods of physiologic stress [31–34], times of adversity (such as famine, drought, and extreme weather aberrations) [35, 36], metamorphosis [37], and immunosuppression [38, 39].

Historically, corticosterone concentrations in vertebrates have been measured using a multitude of sample types including hair, feathers, feces, urine, blood, water, and saliva [40, 41]. Unfortunately, many of the sample types that can be collected in amphibians either measure real-time corticosterone concentrations while inciting a robust response from the HPI, such as blood sampling [42], or measure historic concentrations while only inciting a minor HPI response, such as urine [19]. Corticosterone can be measured noninvasively from amphibians by collecting samples from the surrounding water that they are in, but that requires the animal to be contained in water for 2 hours for accurate interpretation [43]. When collecting urine, it can take up to 2 hours for samples to be collected [20, 44–46]. By the 2-hour mark, the urine that is collected will likely reveal elevated corticosterone concentration due to the diffusion of corticosterone from the blood into the bladder from handling [19]. Inversely, blood samples can capture corticosterone concentrations accurately at a single point in time; however, it is invasive and incites an almost immediate increase in circulating glucocorticoid metabolites [47, 48]. A new method of sample collection has been developed and validated for at least 11 amphibian species involving the use of swabs to collect dermal secretions for corticosterone measurements [19, 49, 50]. This noninvasive and rapid technique has proven to be very effective in measuring glucocorticoids in small amphibians [19, 49, 50].

Marine toads have been the subject of many recent studies because they are an invasive species in many of the ecosystems they inhabit [51, 52]. This means that while contributing to depopulating efforts, researchers can use marine toads as a model for measuring environmental health. Researchers have validated urinary corticosterone assays to measure corticosterone changes in this species [19, 46, 53]; however, the use of dermal swab samples has not yet been validated. The objectives of this pilot study were to (1) measure the average dermal secretion of corticosterone concentration in free-ranging marine toads and to evaluate the changes following transportation and housing in human care for 62 days and (2) compare corticosterone concentrations in dermal secretions and urinary samples of marine toads while in human care.

## 2. Materials and Methods

### 2.1. Animals

This study was performed with approval from the NC State University Institutional Animal Care and Use Committee (IACUC) (IACUC #20-207), the Zoo Miami Animal Care and Use Committee, and the North Carolina Zoo (NC Zoo) research review board.

Sixty-six wild marine toads were caught during the night of August 19, 2020, from the grounds of Zoo Miami in Miami, Florida, USA for a routine invasive species population control program. All the toads were randomly collected and were captured on a first-seen basis. The first ten toads collected (average weight of 128.7 g) were immediately anesthetized with tricaine methanesulfonate (MS-222, Western Chemical Inc.) (10 g/L, buffered with sodium bicarbonate) and then euthanized via anesthetic (MS-222) overdose by a veterinarian. The attending veterinarian confirmed euthanasia using a Doppler ultrasound. The remaining 56 animals were examined by a veterinarian and deemed healthy on visual inspection. Each toad was equipped with a subcutaneous passive integrated transponder (Biomark®, Boise, ID, USA) for individual identification. The marine toads were shipped to the NC Zoo in Asheboro, NC, USA, the following day.

### 2.2. Housing and Diet

Upon arrival at the NC Zoo, the toads were again deemed healthy upon veterinary inspection. The toads were housed in six waterland (Orange, CA, USA) tubs measuring 178.8 cm × 81.3 cm × 35.6 cm (511 liters) with access to hide boxes and a shallow pool of reconstituted reverse osmosis water with a damp towel as a substrate. Starting on day five the pool water was salinated at 2ppt. Individuals were randomly assigned to a tub using Microsoft Excel's shuffle function (Microsoft Office 2013, Redmond, WA, USA). The tubs were cleaned daily due to waste and leftover food and minimally disinfected (chlorhexidine gluconate 2% solution diluted 1 oz per gallon of water) weekly. Each tub also featured a mesh top to prevent escapes and to partially block the artificial lighting. Animals were kept at an ambient temperature (20.5–27.7°C) with a light cycle from 08:00 to 17:00 hours to correspond with the schedule of the animal husbandry staff with no supplemental heating or cooling. Temperature and humidity readings of the room were recorded daily using an AcuRite® (#219CA, Lake Geneva, WI, USA) indoor temperature and humidity monitor [54].

All individuals were fed a diet routinely used by the NC Zoo, which consisted of gut-loaded adult brown house crickets (*Acheta domesticus*) (Catawba Cricket Hatchery Inc. Charlotte, NC, USA). The crickets were gut-loaded with Hi Calcium Gut Loading Diet (Mazuri®, St. Louis, MO, USA) and offered small, sliced pieces of carrots and sweet potatoes before feeding. The toads were examined daily for any changes in their health status. Toads were euthanized in randomly chosen groups (individuals were randomly assigned using Microsoft Excel's shuffle function) of 14 individuals on days 9, 15, 32, and 62. Toads were anesthetized with MS-222 (10 g/L, buffered with sodium bicarbonate), weighed, and euthanized via pithing by a veterinarian. Following euthanasia, a full necropsy was performed by a board-certified pathologist.

### 2.3. Sample Collection

Individuals were randomly assigned to one of the four euthanasia groups using Microsoft Excel's shuffle function (Microsoft Office 2013, Redmond, WA, USA). Dermal secretion samples were collected from all toads during the initial capture in Miami, upon arrival at the NC Zoo, and on the day of euthanasia. Each dermal secretion sample was obtained immediately upon the animal's capture from the ground for the initial capture, as soon as the animal was removed from the shipping container upon arrival at the NC Zoo and immediately upon each animal's capture from the Waterland tubs on the day of euthanasia. For dermal secretion samples, each animal was swabbed 10 times on both the dorsal and ventral aspects of their bodies using sterile wooden cotton-tipped applicators, which were then placed in Fisherbrand™ cryogenic storage vials (Thermo Fisher Scientific, Waltham, MA, USA) and frozen at −80°C. The collection of each dermal secretion sample took less than 30 seconds after capturing the animal in-hand.

Urine collection was not attempted on any toad on the day of capture due to a COVID-19 restriction policy instituted at Zoo Miami. Due to previous experiences by the authors, urine collection was not attempted on the day of arrival due to the lack of urine production by the majority of the animals. Free-catch urine was collected from 14 toads on day 9, 11 toads on day 15, 12 toads on day 31, and 11 toads on day 62, by holding toads over a clean plastic container until they urinated. If the toads did not urinate immediately, they were placed in the container for up to 10 minutes to urinate. If the urine sample was not obtained within the 10-minute allotted time period, no sample was collected for that animal. At least 0.1 mL of urine was collected into 2.0 mL Fisherbrand™ cryogenic storage vials using a plastic pipette and centrifuged for 3 minutes at 1600 revolutions per minute (415 g). Urine and samples were stored at −80°C until shipment on dry ice to the Smithsonian Conservation Biology Institute (Front Royal, VA, USA).

The dermal secretion samples obtained during the initial capture in Miami were acquired at night, while the samples acquired upon arrival at the NC Zoo were acquired midmorning. Both the dermal secretion and urine samples were obtained midmorning during the subsequent day of euthanasia. The sex was determined during the postmortem necropsy of the individuals.

### 2.4. Urinary and Dermal Secretion Enzyme Immunoassay

Corticosterone concentrations in urine and dermal secretions were analyzed as a double-antibody enzyme immunoassay (EIA) that relied on a polyclonal rabbit anticorticosterone antibody (CJM006) [54]. Samples of high-quality and low-quality controls and standards (3.9–1000 pg/well; Sigma Diagnostics, Inc., Livonia, MI, USA) were added in duplicate (50 *μ*l per well) to precoated goat antirabbit IgG and 96-well plates at an ambient temperature. Corticosterone-horseradish peroxidase (25 *μ*l, 1 : 20,000 dilution; SCBI, Front Royal, VA, USA) was then added to all the wells followed by an anticorticosterone antibody (25 *μ*l, 1 : 60,000 dilution) to all the wells except the nonspecific binding wells. Microplate sealers were placed over the plates and incubated at an ambient temperature on an agitator (Model E6121; Eberbach Corp., Ann Arbor, MI, USA) for 1 hour. The plates were then washed 4 times with wash buffer (1 : 20 dilution, 20X Wash Buffer Cat. No. X007; Arbor Assays, Ann Arbor, MI, USA), and thoroughly dried before adding 3, 3′, 5, 5′-tetramethylbenzidine (TMB) to the wells (100 *μ*l, Moss Inc., Pasadena, MD, USA) and incubating for 30–45 minutes without agitation at an ambient temperature. To halt the reaction, 50 *μ*L of a 1 N HCl solution was added to all wells and optical density was read from a plate reader at 450 nm (OPsys MR; Dynex Technologies, Chantilly, VA, USA). The CMJ006 antibody was validated for the use with marine toad urine and dermal secretion samples by showing parallelism between sample dilutions and the standard curve, and significant recovery of exogenous corticosterone added to samples before analysis (urine: *y* = 1.180*x* − 3.603, *R*^2^ = 0.995; dermal swab: *y* = 1.271*x* + 7.502, *R*^2^ = 0.998). To account for urine concentration, USpG was used as an index for corticosterone concentrations in urine samples using a published correction factor as follows: USpG = raw corticosterone concentration (ng/mL) times (USpG average of all samples − 1)/(USpG sample − 1) [35, 47]. The assay sensitivity was 0.14 ng/mL (based on 90% binding) and interassay and intraassay CVs were <15% and 10%, respectively. Any samples with duplicate CVs >10% were reanalyzed.

### 2.5. Statistical Analysis

Data were analyzed using JMP Pro, version 16.0 software (SAS Institute Inc., NC, USA). All the variables were tested for normality of distribution by the Shapiro–Wilk test. Outliers were identified and tested utilizing the interquartile rule (1.5 ∗ IQR) for the population as a whole and for each group stratified. Data points had to be outliers for the entire population and for each stratum before them being removed so as not to erroneously remove extreme values. A Pearson correlation coefficient was computed to assess the relationship between the corticosterone concentration in urine samples and dermal swabs at each of the different euthanasia time points as well as the relationship between the weight of the animals and the corticosterone concentration in samples. Overall differences in corticosterone concentrations between sample types (urine vs. dermal secretions) and sex were assessed using a Wilcoxon rank-sum test. Differences in corticosterone concentrations between each of the sample collection time points were assessed using a Kruskal–Wallis test, followed by a Steel–Dwass all-pairs multiple comparison test with Bonferroni correction applied. Values were reported as mean ± SEM, median, minimum, and maximum and were considered to be significant at *P* ≤ 0.05.

## 3. Results

Of the 66 toads collected for this study, 42 were males, 22 were females, and 2 were unknown. Toad weights at the time of euthanasia ranged from 50.0 to 247.0 g (mean: 144.2 g). The exact age of the animals was unknown, but all were phenotypically subadults to adults. Five dermal samples were excluded from the day of capture and one sample was excluded from day 9 because there was not sufficient volume to be analyzed.

There was a weak negative correlation observed between corticosterone concentrations reported in dermal swabs and those in urine samples at all four of the euthanasia time points (day 9: r(11) = −0.26, *P* = 0.38; day 15: r(9) = −0.012, *P* = 0.71; day 32: r(10) = −0.29, *P* = 0.36; day 62: r(9) = −0.22, *P* = 0.51). There was also a weak positive correlation noted between corticosterone concentrations reported in the dermal swabs and the weight of the toads (r(53) = −0.11, *P* = 0.38) and a weak negative correlation observed between corticosterone concentrations reported in the urine samples and the weight of the toads (r(45) = 0.05, *P* = 0.73).

No significant difference in corticosterone concentrations was detected between the toad sexes (swabs: *P* = 0.15, urine: *P* = 0.66); therefore, all the data were combined (Table 1). There was a significant difference (*P* ≤ 0.05) in mean urine corticosterone concentrations in urine samples between day 9 (0.45 ± 0.07 ng/mL) and day 32 (0.21 ± 0.06 ng/mL). Dermal swabbing proved to be an effective method for measuring corticosterone in marine toads. Corticosterone concentrations were significantly higher (*P* ≤ 0.05) on the day of capture (0.64 ± 0.03 ng/mL) and the day of arrival (0.67 ± 0.03 ng/mL) than on day 15 (0.47 ± 0.03 ng/mL). When comparing the urine corticosterone concentration with the dermal secretion corticosterone concentration from the same sample day, only samples on day 15 were significantly different (*P* ≤ 0.05) (urine: 0.21 ± 0.06 ng/mL; dermal swab: 0.52 ± 0.04 ng/mL) (Table 1).

## 4. Discussion

The limited data presented in this study highlight that while both urine and dermal secretions can be used to measure corticosterone concentrations in marine toads and appear to follow similar trends, the two methods are not directly comparable since the numerical results are different. This difference in values is likely due to the time that it takes for corticosterone concentration changes to be detected in the different sample types. Previous research demonstrated that it can take 2 hours to see elevations in corticosterone concentrations in urine after a stressful event, such as transportation [45]. Comparatively, Romero and Reed found in their investigation of corticosterone concentration in reptiles and birds that increased concentrations can be measured in less than 3 minutes in blood samples after handling [42]. As for dermal secretions, researchers found that increases in corticosterone concentrations can be detected in species such as the green treefrog (*Hyla cinerea*) and red-spotted newt (*Notophthalamus viridescens*) immediately after a stressor, such as a manual restraint for 5 minutes or a ACTH injection [49]. It is documented that changes in corticosterone concentrations can continue oscillating for up to 2 hours after a stressor in the dermal secretions of multiple amphibian species [18, 49, 50].

Unfortunately, we are unable to corroborate the numerical values with some of the published literature on marine toads because the values reported are in different units (pg/*μ*g) [20, 23, 45]. However, there are published data on the corticosterone concentrations of 10 other amphibian species with comparable units (mg/mL) [19, 49]. The ranges reported in this study are similar to prestressed concentrations reported in Green treefrogs (*Hyla cinerea*), American toads (*Anaxyrus americanus*), and red-spotted newts (*Notophthalamus viridescens*) in a study where the ACTH stimulation was conducted on 9 terrestrial and aquatic amphibian species [49] and in a second study that examined the stress response of Wyoming toads (*Anaxyrus baxteri*) to ACTH stimulation. It should be noted that it is possible that the values of the corticosterone levels reported here as well as in the previous literature are a combination of corticosterone as well as their metabolites. This is one fault in our technique that is a product of the sensitivity of the assays that we have available to us, and the tests that were conducted on the samples. Future studies should include collecting blood and fecal samples in order to corroborate levels between different sample types and differentiate between the various metabolites of corticosterone.

The data collected from this study suggest that mean corticosterone concentrations in the dermal secretions obtained from dermal swabs of marine toads in human care are less than the mean concentrations of free-ranging marine toads immediately after capture and after transportation. This could not be corroborated with the urine samples because urine collection was not attempted on any toad on the day of capture due to a COVID-19 restriction policy instituted by Zoo Miami. Collection was also not attempted post-shipment due to the poor collection rate following the capture and shipment of these animals in a previous study by the authors [55]. Although the dermal samples from day 15 are the only ones statistically different from those on the day of capture and day of arrival, the mean, median, and highest individual corticosterone concentrations were all visually less than the respective comparable parameters from the capture and arrival dates. Previous work measuring urinary corticosterone concentrations in wild-caught marine toads also demonstrated that there is a significant decrease of corticosterone in toads under human care versus free-range [20, 45, 46]. It is postulated that this decrease is due to the stressful variables, such as finding a mate and food availability being mitigated in human-care environments [46]. The lack of an observable statistical difference in corticosterone concentration in the dermal samples of free-ranging toads and those in human care may be the results of our study design. There is research that suggests amphibians can experience an increase in corticosterone concentrations just by perceiving a threat such as a predator [34] and it only takes 3 minutes for elevations in corticosterone concentrations to be detected in samples that are more directly associated with blood [39, 42], such as dermal secretions [49]. It is possible that the toads experienced a release of corticosterone as soon as they saw a human, or in response to another toad being removed from their shared enclosure. Future work could examine long-term corticosterone concentration trends in marine toads using serial dermal secretions to better test this hypothesis.

Studying the stress response in animals is complex due to the multitude of variables that must be accounted for and the variety of physiologic systems that it affects [28, 30, 32, 33]. The association of zoos and aquariums (AZA) has developed almost 500 species survival plans (SSP) that manage *ex-situ* populations in accredited zoos and aquariums [56]. However, studies have demonstrated that long-term increases in corticosterone concentrations decrease circulating testosterone levels which lead to a decreased sperm production and a decrease in mating behaviors [9, 22, 57–59]. One purpose of this study was to determine if dermal swab samples could be used to measure corticosterone concentrations in marine toads, which our data indicated that it could be. Since this study was focused on determining if dermal swabs were effective at obtaining clinically useful samples, key variables that are known to modulate corticosterone concentrations, such as ambient temperature [23], circadian rhythm [27], and substrate [60], were not regulated. In addition, we did not measure circulating leukocytes or the presence of disease, which can augment corticosterone in certain species of amphibians, but not others [38]. Further work can be performed to evaluate and modulate the artificial habitats that amphibians are kept in and the husbandry they receive under human care to assess the impact these variables have on baseline corticosterone concentrations.

Our data did indicate that there was no correlation between toad weight and corticosterone concentration, which has been demonstrated in previous studies with marine toads as well [46]. No statistical difference in corticosterone concentrations was observed between the sexes of the toads. Previous research has shown that testosterone concentrations are correlated with corticosterone concentrations in amphibians [30]; however, there has yet to be a dedicated study that examines and compares the trends of corticosterone concentrations through a breeding season between male and female amphibians. These findings differ from the work performed on avian and mammalian species, which show differences in cortisol levels depending on sex, weight, and diet [61–64].

Research conducted in a laboratory setting found that marine toads that were subject to a standard thermal shock regime of 30 minutes exposed to a water bath maintained at 35°C for 34 days had increasing corticosterone levels compared to the control group, which was kept at a controlled ambient temperature of 25°C [23]. The marine toads in this study were collected from Miami, FL, USA, where the mean temperature was 29.8°C for the month of August, which was the warmest August on record [65]. The temperature range for the day of capture was 27.7–33.8°C [66]. While in human care, the marine toads were housed between 20.5 and 27.7°C. It is possible that the transition from the warm climate in Florida to the cooler climate of the NC Zoo may have contributed to the difference in corticosterone concentrations between free-ranging animals and those in human care.

While studying the effects of artificial light at night (ALAN) on marine toads, researchers found reduced saliva corticosterone concentrations and crepuscular activity levels in individuals who were kept in a high-light enclosure during the night [27]. It was proposed that this change was due to a change in activity periods and that corticosterone peaks occurred when there was a spike in activity. However, the researchers only collected salivary samples on the first and last day of the experiment in the morning, so they were not able to substantiate the proposal with their data [27]. In this study, at their date of capture the marine toads were acclimated to 13 hours of sunlight from 06:50 to 19:58 [67]. To align with the times of the staff, the toads received fluorescent light from 08:00 to 17:00. Although samples were collected roughly during the same time of the day, it is possible that the toads were in the process of adapting to the new light schedule in human care, thereby skewing the data collected over the course of the study. This is a complex variable because although the type and duration of light were changed going from free-range to human care, it is possible that the toads may have been previously stressed by the significant amount of light pollution while being free-range in Miami, Florida [68]. In addition, the initial dermal swab samples were collected at night when the toads were captured whereas the subsequent samples were taken in the morning. This factor may have augmented the data since corticosterone concentration fluctuates during the day and is the highest during the morning hours when the toads are the most active [27]. The relationship between corticosterone concentrations and photoperiod could be further explored in amphibians in human care by serially measuring corticosterone levels at different times of the day.

Research has also been conducted on habitat preference in the common toad (*Bufo bufo*) and found that adults kept on a plowed soil had higher corticosterone concentrations than adults kept on the meadow or forest litter [68]. As for juvenile toads, there was not a measurable change in corticosterone concentrations between the three substrate types. They proposed that this change could be due to adult toads experiencing a variety of terrain types and preferring terrains that were more common in their environment while juveniles were unfamiliar with all the terrain selections because they were only used to water [68]. The marine toads were collected from across the 750-acre grounds of the Zoo Miami where substrates ranged from forest litter to concrete enclosures. In stark contrast, the toads were housed on a damp towel to aid in the daily cleaning of the enclosure while in human care. This is likely a variable that augmented the corticosterone concentrations in this study.

Marine toads have been a recent subject of study because they allow researchers to study the physiologic changes when an invasive species colonizes a new ecosystem. Ever since their introduction to Australia in 1935, marine toads have been a threat to both native wildlife and the domestic species that reside there. Researchers have taken advantage of their invasive nature to study how stress levels and immunity work simultaneously [69, 70]. They found that toads that were captured at an invasive front had a positive correlation between corticosterone concentration and increased leukocyte oxidative burst, the mechanism by which leukocytes rapidly release reactive oxygen intermediates during the phagocytosis of microbes. Interestingly, in the same study, researchers did not find a correlation between a toad's burden of nematode lungworms (*Rhabdias pesudosphaerocephala*) and corticosterone concentrations [69]. Additional research on the parasite burden of marine toads shows that toads that live along invasion fronts have more parasites than the native amphibian species in the same geographic region [70]. Inversely, in studies measuring the immune response of marine toads in Australia, researchers found that toads traveling at the fringe of their territory were not able to fight an *E. coli* infection as well as their neighbors that remained well within their territory [70]. These conflicting data demonstrate how confounding measures of stress and immunocompetency in a single species can be despite their close association.

Researchers have been attempting to relate corticosterone concentrations and infection with the fungal pathogen *Batrachochytrium dendrobatidis* (Bd), which has been the cause of the global extinction of amphibian species for over 20 years [71]. A collection of studies has described the relationship in 13 species across different life stages; however, there is no single resounding conclusion [72]. The toads in this study were not tested for Bd, so there was no way to know if any of the toads were infected with it. However, it is unlikely because adult marine toads are infrequently infected by Bd in large part due to the unique microbiome of their skin [73, 74].

## 5. Conclusion

This study paves the way for future work to validate the dermal swab method for obtaining samples for measuring dermal secretion corticosteroid concentrations in marine toads. In addition, this research demonstrates the utility of combining two methods for assessing the adrenal activity to obtain a more complete picture of chronic and acute stressors in amphibians.

## Figures and Tables

**Table 1:** A comparison of the mean concentration of corticosterone in both male and female marine toads (*Rhinella marina*) from urine and dermal secretions sampled at capture, immediately following a 16-hour transport from Miami FL to Asheboro, NC and 4 time points over 60 days in human care at the North Carolina Zoo.

Day of sampling	Urine corticosterone (ng/mL)				Dermal secretion corticosterone (ng/mL)			
	*N*	Mean ± SEM	Median	(Min, max)	*N*	Mean ± SEM	Median	(Min, max)
Capture day		N/A	N/A	N/A	61	0.64 ± 0.03^a^	0.62	(0.31, 1.78)
Arrival day		N/A	N/A	N/A	56	0.67 ± 0.03^a^	0.62	(0.22, 1.31)
Day 9	14	0.45 ± 0.07^a,*ψ*^	0.47	(0.04, 0.87)	13	0.51 ± 0.05^a,b,*ψ*^	0.49	(0.25, 0.98)
Day 15	11	0.37 ± 0.07^a,b,*ψ*^	0.41	(0.03, 0.70)	14	0.47 ± 0.03^b,*ψ*^	0.45	(0.28, 0.74)
Day 32	12	0.21 ± 0.06^b,*ψ*^	0.13	(0.03, 0.78)	14	0.52 ± 0.04^a,b,Φ^	0.50	(0.28, 0.89)
Day 62	11	0.31 ± 0.07^a,b,*ψ*^	0.25	(0.10, 0.93)	14	0.54 ± 0.04^a,b,*ψ*^	0.50	(0.23, 0.85)

N/A = not available. ^a,b^Within a column, differing superscripts indicate urine or dermal corticosterone differences by date (*P* < 0.05). ^*ψ*,Φ^Within a row, differing superscripts indicate urine vs dermal corticosterone differences on the same date (*P* < 0.05).

## Data Availability

The data used to support the findings of the study are available from the corresponding author upon request.
